# Effect of Trace-Ti Content on Microstructure and Mechanical Properties of 1Cr10Co6MoVNbN Steel

**DOI:** 10.3390/ma19173695

**Published:** 2026-08-30

**Authors:** Guoxin Hu, Chenguang Shang, Tingyao Liu, Aobo Du, Huaibei Zheng, Yonghao Lu

**Affiliations:** 1 National Center for Materials Service Safety, University of Science and Technology Beijing, Beijing 100083, China; a934246947@163.com (G.H.); chenguangshang@163.com (C.S.); 15512470791@163.com (A.D.); 2 Chengdu Advanced Metal Materials Industrial Technology Institute Co., Ltd., Chengdu 610300, China; liutingyao8211371@163.com (T.L.);

**Keywords:** martensitic stainless, 1Cr10Co6MoVNbN steel, Ti content, mechanical property, precipitate

## Abstract

The effect of Ti content on the microstructure and mechanical properties of 1Cr10Co6MoVNbN steel was systematically investigated by comparing a low-Ti steel (LTS, 0.0066 wt.%) with a high-Ti steel (HTS, 0.02 wt.%). Increasing the Ti content was found to markedly alter the precipitate distribution and, consequently, the creep and high-temperature tensile behavior. In HTS, the higher Ti level promoted the preferential formation of (Ti,Nb)N inclusions during solidification; EDS analyses indicated that the matrix of HTS was substantially depleted of Nb, N, Mo, and V relative to LTS, consistent with this preferential nitride formation. In the tempered state, HTS exhibited larger NbC particles that were frequently attached to (Ti,Nb)N inclusions, whereas LTS contained finer, more uniformly dispersed NbC and abundant needle-like Cr_2_N precipitates within martensitic laths. These microstructural differences correlated with a substantially higher 100 h creep strain in HTS compared with LTS at 550 °C and 325 MPa, and with a 550 °C tensile strength in HTS that fell below the aerospace standard requirement. Post-creep examination further revealed void formation around (Ti,Nb)N inclusions in HTS, suggesting that these inclusions act as stress concentrators that reduce the effective load-bearing area during creep. The results indicate that strict control of Ti content is essential in Nb–N-strengthened martensitic steels to avoid excessive (Ti,Nb)N formation and the associated degradation of high-temperature mechanical performance.

## 1. Introduction

1Cr10Co6MoVNbN, which is similar to Jethete M152, is a martensitic heat-resistant stainless steel, used for rotors, blades, disks and seal rings in high-pressure compressors of aircraft engines due to its high thermal strength, excellent creep resistance, and low notch sensitivity [1,2,3]. Its excellent properties lie in its microstructure of multi-scale synergistic strengthening, including martensitic laths with high dislocation density and a variety of precipitates. Uniformly dispersed nanoscale MX-type carbonitrides effectively pin dislocations through the Orowan strengthening mechanism, while M_23_C_6_ carbides hinder grain boundary sliding at high temperatures, significantly enhancing creep resistance [4]. The type and quantity of precipitated phases are not only affected by the heat treatment, but also by the alloy elements, such as Ti, Al, V and N [5,6,7,8,9,10,11,12,13]. An increase in Al would reduce the content of free N in the steel, and a large amount of Al, AlN preferentially formed, would reduce the available N for forming MX carbonitrides, which could hinder dislocation movement and a stabilized substructure to improve creep strength and make the number density of MX insufficient to reduce creep strength [5,6,7]. For N-strengthened steels, N/Al ≥ 4 is recommended to prevent excessive consumption of N by Al [6,14]. The increase in Ti content could promote the formation of residual ferrite and improve creep strength [10], or form a fine MX phase to improve creep strength [9,13]. Although there has been considerable research on the effects of N-strengthened steels or Ti content on the mechanical properties of steel, the influence mechanism of Ti elements on N-strengthened steels still lacks a systematic investigation. For 1Cr10Co6MoVNbN steel in industrial application, the specific lower and upper bounds to which Ti content should be controlled in order to meet the mechanical property requirements specified in the aerospace material standard WS 9-6521-78 have not yet been systematically established.

The primary objective of this study is to investigate the effect of trace Ti content on the microstructural evolution and mechanical properties of 1Cr10Co6MoVNbN martensitic heat-resistant steel, and to elucidate the underlying correlation between Ti content, precipitate characteristics, and creep/high-temperature tensile performance. Specifically, by comparing steels with low Ti content (0.0066 wt.%) and high Ti content (0.02 wt.%), this work aims to (1) clarify how Ti content influences the formation of (Ti,Nb)N inclusions and the consequent depletion of strengthening elements (Nb, N, Mo, V) in the matrix; (2) reveal the resulting changes in precipitate types, sizes, and distributions (e.g., NbC, Cr_2_N); and (3) establish how these microstructural variations govern the material’s solid solution strengthening, dispersion strengthening, and creep resistance, thereby providing a quantitative basis for optimizing Ti control in industrial production.

## 2. Materials and Methods

**Figure 1:**
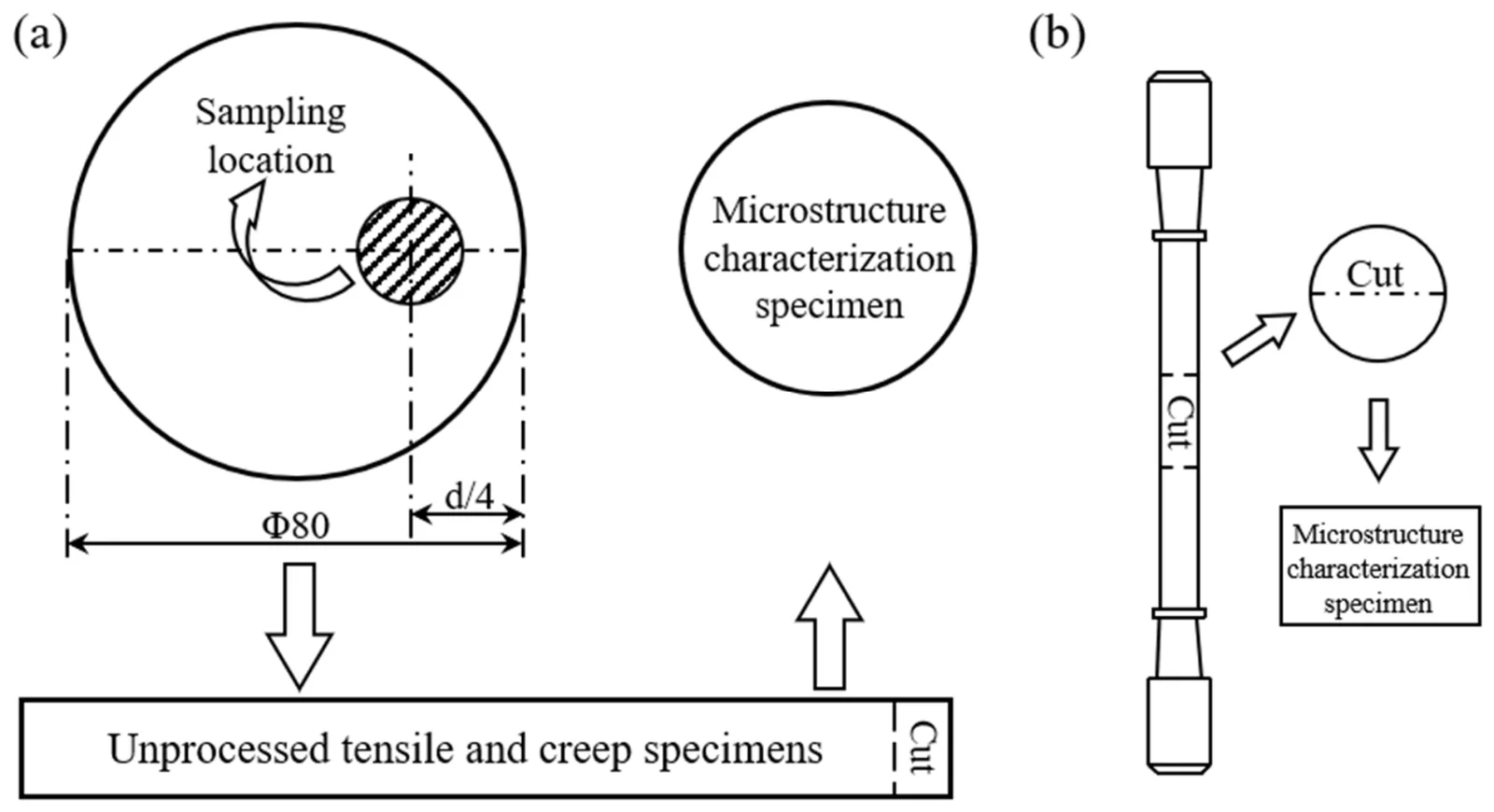
Sampling diagrams: (**a**) tensile and creep specimens, along with the sampling method for microstructure characterization before creep; (**b**) sampling method for microstructure characterization after creep.

In this study, 1Cr10Co6MoVNbN(1Cr10) steels with two different Ti contents were investigated, which were manufactured by Pangang Group Company Ltd., Panzhihua, Sichuan, China. Both alloys were synthesized by vacuum induction melting (VIM) followed by electroslag remelting (ESR). The base charge materials supplied by Pangang Group Company Ltd. (Panzhihua, China), consisted of high-purity iron, cobalt, chromium, molybdenum, vanadium, and niobium, with carbon added as graphite. Nitrogen was introduced via ferrochrome nitrides. The two Ti levels were achieved by adding different amounts of a ferrotitanium (Fe–Ti) master alloy to the melt during the VIM stage. After homogenization, the ingots were forged and hot-rolled into Φ80 mm round bars. Specimens for heat treatment were taken from positions near the centerline of the homogenized bars. Their chemical compositions were determined according to the GB/T 223 series standard [15] methods for chemical analysis of iron, steel, and alloy (Standard Methods for Chemical Analysis of Iron, Steel and Alloy), and the results are listed in Table 1. The 1Cr10 steel with low Ti content is referred to as “LTS”, while that with high Ti content is referred to as “HTS”. The heat treatment consisted of austenitizing at 1170 °C for 45 min followed by oil quenching, and double tempering at 620 °C for 2 h each with air cooling between stages. All microstructural analyses and mechanical tests reported in this study were performed on quenched and tempered specimens of both LTS and HTS. After heat treatment, small sections were cut from the front ends of the rod bars, as shown in Figure 1a, for microstructure characterization. Room-temperature tensile tests were conducted on specimens with a gauge diameter of 10 mm and a gauge length of 60 mm using a CMT5205 universal testing machine at a constant strain rate of 0.00025 s^−1^ in accordance with GB/T 228.1-2021 [16]. High-temperature tensile tests were performed on specimens of the same geometry using an E45.105 system at 550 °C, also at a constant strain rate of 0.00025 s^−1^ in accordance with GB/T 228.2-2015 [17]. Creep tests were carried out on specimens with a gauge diameter of 10 mm and a gauge length of 50 mm using an RDL50 kN high-temperature creep testing machine at 550 °C and 325 MPa for 100 h. At least three replicate tests were performed for each mechanical test condition.

The microstructures of the investigated samples before and after creep were characterized using a Zeiss Merlin scanning electron microscope (SEM) equipped with an energy-dispersive X-ray spectroscopy (EDS) system, employing secondary electron imaging at an acceleration voltage of 15 kV. EDS point analyses were performed on 2 randomly selected regions per material condition, deliberately avoiding (Ti,Nb)N inclusions and targeting relatively inclusion-free areas. In addition, EDS elemental mapping was conducted on 3 representative (Ti,Nb)N inclusions in HTS to confirm the composite structure.

To further analyze the changes in precipitates before and after creep deformation, the specimens shown in Figure 1b were cut into small pieces of Φ5 mm × 0.5 mm, ground with sandpaper to a thickness of less than 100 μm, and subsequently thinned by argon ion milling (Gatan PIPS, 4 kV Ar^+^ ions, incident angle 4–6°, until perforation) for transmission electron microscopy (TEM) analysis. TEM observation was conducted using a FEI Tecnai G2 F20 transmission electron microscope. Bright-field (BF), dark-field (DF), and high-resolution (HRTEM) images were recorded from at least 2 distinct lath regions per foil to ensure representative microstructural observation. Selected-area diffraction (SAD) patterns were acquired from 3 different precipitate locations (needle-like, granular, and lath-boundary phases) to verify phase identity. Selected-area diffraction (SAD) patterns were acquired from circled regions in the corresponding TEM images using a selected-area aperture of 100 nm.

Statistical analysis of non-metallic inclusions was performed with an ASPEX inclusion analysis, quantifying the primary types, quantities, number, and size distribution of inclusions over a total analyzed area of 162 mm^2^. The 162 mm^2^ area was chosen to ensure statistical representativeness, particularly for the relatively large (Ti,Nb)N inclusions (average size ~2–5 μm) that are sparsely distributed in LTS.

In addition, a SmartLab 9kW X-ray diffractometer (Rigaku Corporation, Akishima, Tokyo, Japan; Cu Kα, λ = 0.15406 nm) was employed to determine the dislocation densities of the materials before and after creep. The dislocation density was calculated from the X-ray diffraction peak broadening using the modified Williamson–Hall method. The integral breadths of at least five (hkl) reflections ((110), (200), (211), (220), and (310)) were measured and fitted with a pseudo-Voigt function after background subtraction and Kα_2_ stripping using Origin 2026a. The instrumental broadening was determined using a standard LaB_6_ sample and subtracted quadratically from the measured peak breadths. The modified Williamson–Hall equation was applied:

(1) $$\beta^{*}=\frac{0.9}{D}+K\cdot M\cdot \sqrt{\rho }\cdot b\cdot \frac{\pi }{2}\cdot \frac{\sin\theta }{\lambda }$$

where β* = *β · cosθ*/*λ* is the modified integral breadth, D is the effective crystallite size, K is a constant (average value of 3, corresponding to a mixed screw/edge character), M is a dislocation arrangement parameter (taken as 1 for a random distribution), ρ is the dislocation density, and b is the magnitude of the Burgers vector (b = a√3/2 ≈ 0.248 nm for the BCC martensite with lattice parameter a ≈ 0.287 nm). The contribution of twin faults and stacking faults to peak broadening was assumed negligible in this BCC steel. This method yields the total dislocation density, which predominantly reflects the mobile dislocation population relevant to creep deformation.

During the preparation of this manuscript, the authors used Kimi (Moonshot AI, version 2024) to assist in the formatting and refinement of figures and graphical elements. The authors have reviewed and edited all outputs generated by this tool and take full responsibility for the content of this publication.

## 3. Results

**Figure 2:**
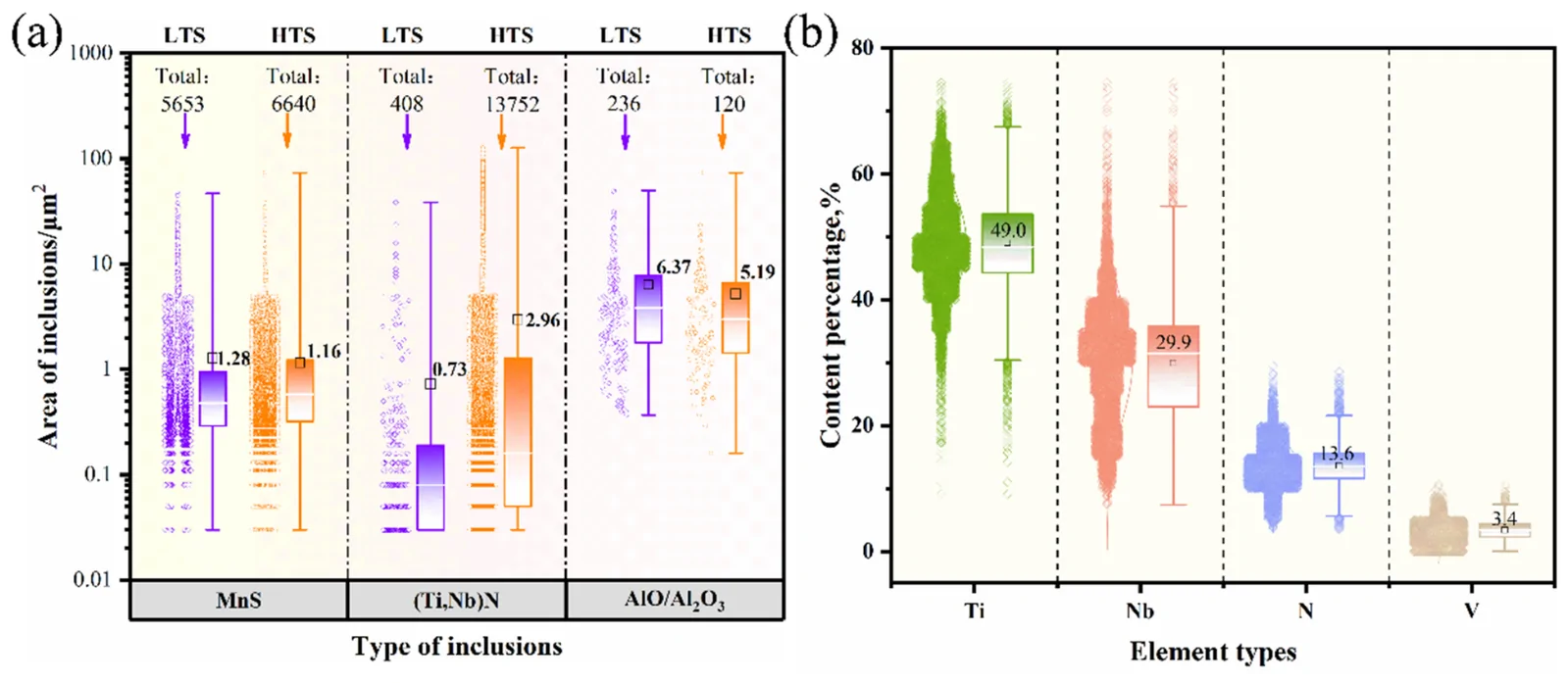
Main types, quantities, and size distribution of inclusions in LTS and HTS steels: (**a**) size distribution of major inclusions; (**b**) elemental composition of (Ti,Nb)N inclusions.

Figure 2 shows the statistical results of inclusions in the two materials. It is observed that the number of (Ti,Nb)N inclusions in HTS is significantly higher than that in LTS. The number density of (Ti,Nb)N inclusions in HTS reaches 94 per mm^2^, whereas in LTS it is only 4.5 per mm^2^, representing a 21-fold increase. The average size of (Ti,Nb)N inclusions in HTS is 2.23 μm larger than that in LTS, and their size distribution range is also broader. In addition to (Ti,Nb)N inclusions, other types such as MnS and Al_2_O_3_ are observed in both materials, but not notable difference in quantity or size distribution of these inclusions are detected between the two materials. For the (Ti,Nb)N inclusions in HTS, ASPEX was used to perform quantitative elemental analysis on over 10,000 particles. As shown in Figure 2b, these inclusions primarily enriched Ti, Nb and N, with a minor enrichment of V.

**Figure 3:**
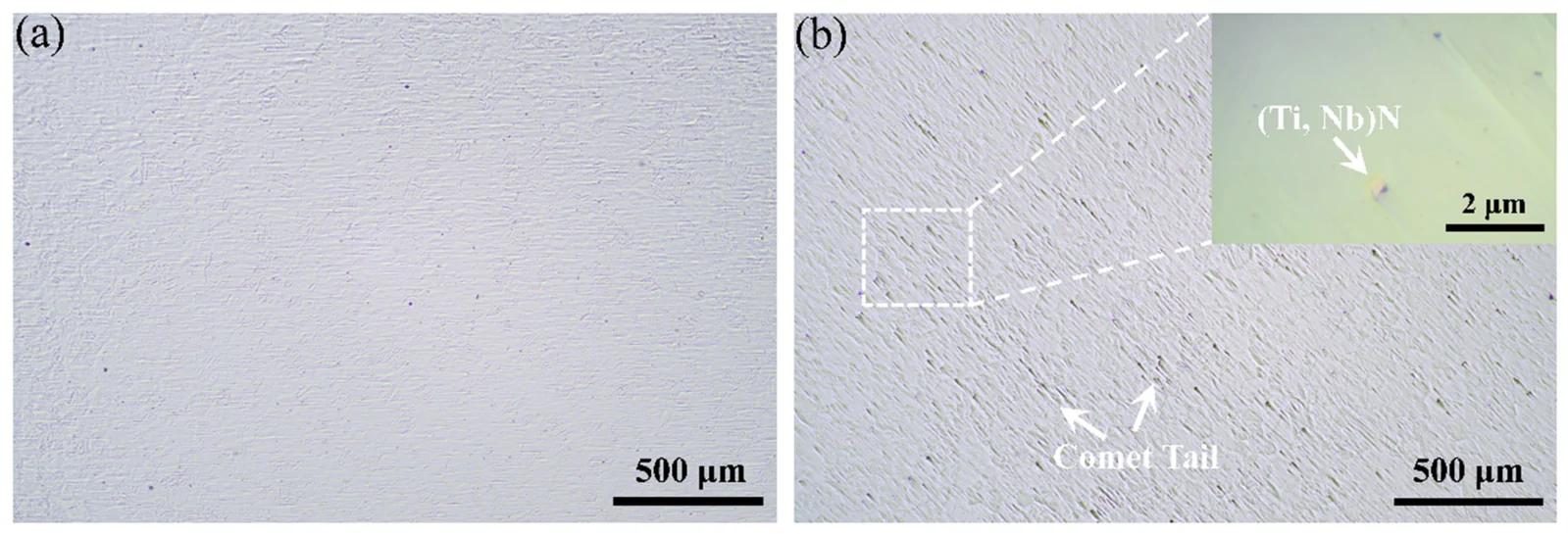
Optical micrographs of LTS/HTS after polishing: (**a**) LTS; (**b**) HTS.

To more intuitively observe the difference in the number of (Ti,Nb)N inclusions between the two samples, both were polished and examined using an optical microscope (OM). Figure 3 shows the OM images of the microstructures in LTS and HTS after polishing. It is observed that the polished surface of LTS is relatively smooth with no apparent inclusions, whereas the surface of HTS exhibited numerous hard black dots, which are presumed to be (Ti,Nb)N inclusions. These inclusions are believed to be harder than the base metal, resulting in the formation of ‘comet tails’ during polishing. Upon magnification, there black dots are revealed to be angular golden structures, as shown in the inserted in Figure 3b.

**Figure 4:**
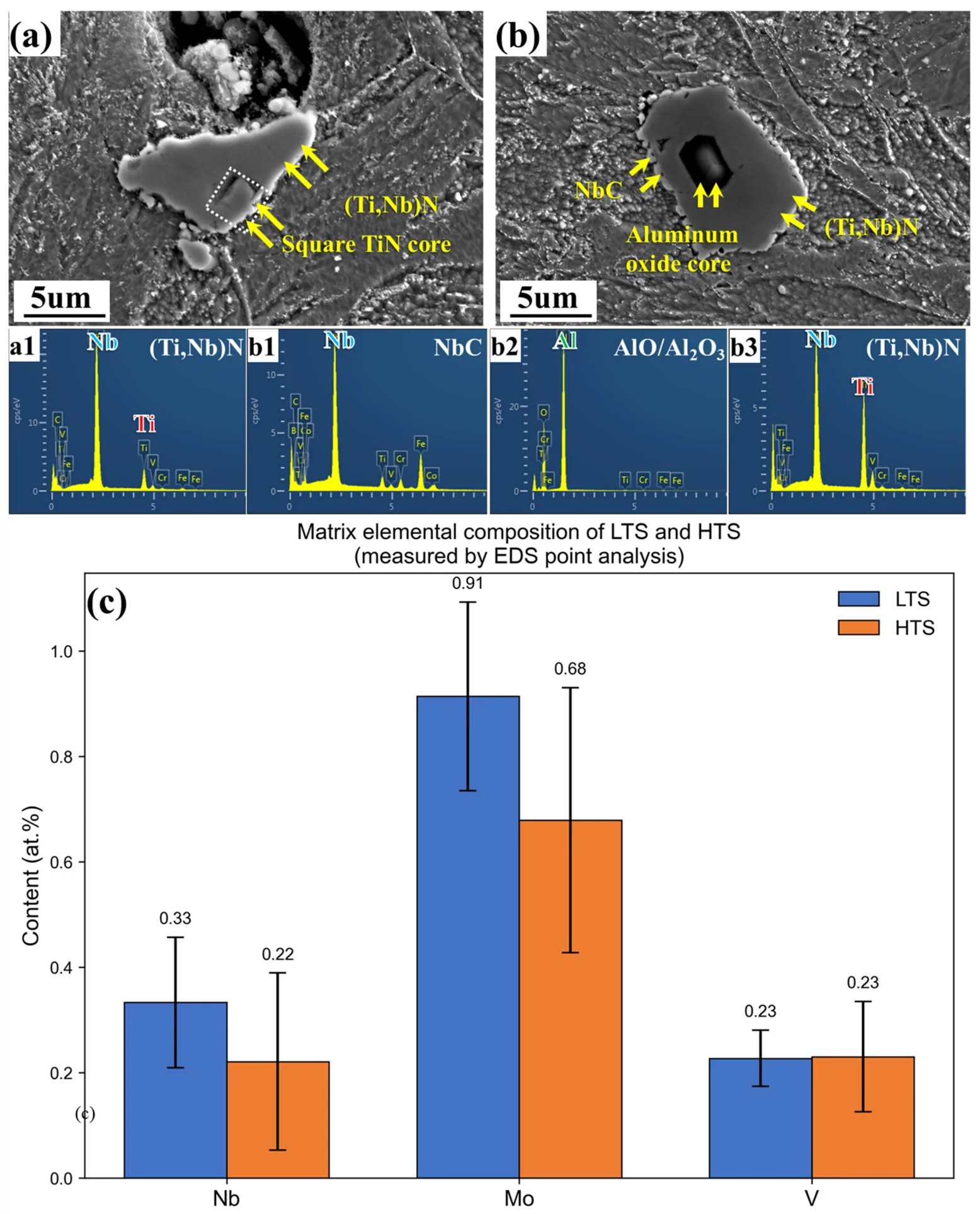
(Ti,Nb)N composite inclusions: (**a**) TiN core (Ti, Nb)N segregation, with (**a1**) corresponding EDS point analysis; (**b**) primary NbC particle-dependent alumina core (Ti,Nb)N inclusions, with (**b1**–**b3**) corresponding EDS point analyses; and (**c**) average matrix concentrations of Nb, Mo, and V in LTS and HTS, with error bars representing the standard deviation calculated from the 10 randomly selected EDS point analyses.

Figure 4 shows the SEM image and corresponding EDS elemental maps illustrating the morphology of (Ti,Nb)N composite inclusions in the HTS. These inclusions are observed in two typical forms: (Ti,Nb)N inclusions with alumina as the core, surrounded by primary NbC and (Ti,Nb)N inclusion ns with square TiN as the core. During the solidification of molten steel, the formation of a large number of inclusions enriched in Nb, N, and V inevitably leads to a general depletion elements in the matrix. This phenomenon is confirmed by EDS mapping on 10 randomly selected regions of the LTS and HTS, deliberately avoiding (Ti,Nb)N inclusions and targeting relatively inclusion-free areas. The results, summarized in Table 2 and Figure 4c, indicate that the Nb content, substantially enriched within (Ti,Nb)N inclusions, is significantly higher in the LTS matrix than that in the HTS matrix. Previous studies have reported that the Nb plays a crucial role in enhancing the short-term creep performance of steels [18]. Consequently, the overall reduction of Nb in the matrix is expected to exert an adverse effect on the short-term creep resistance of the material. It should be noted that the reduction in matrix N content is inferred from the decreased Cr_2_N precipitation, as direct EDS quantification of N is unreliable due to its low atomic number.

**Figure 5:**
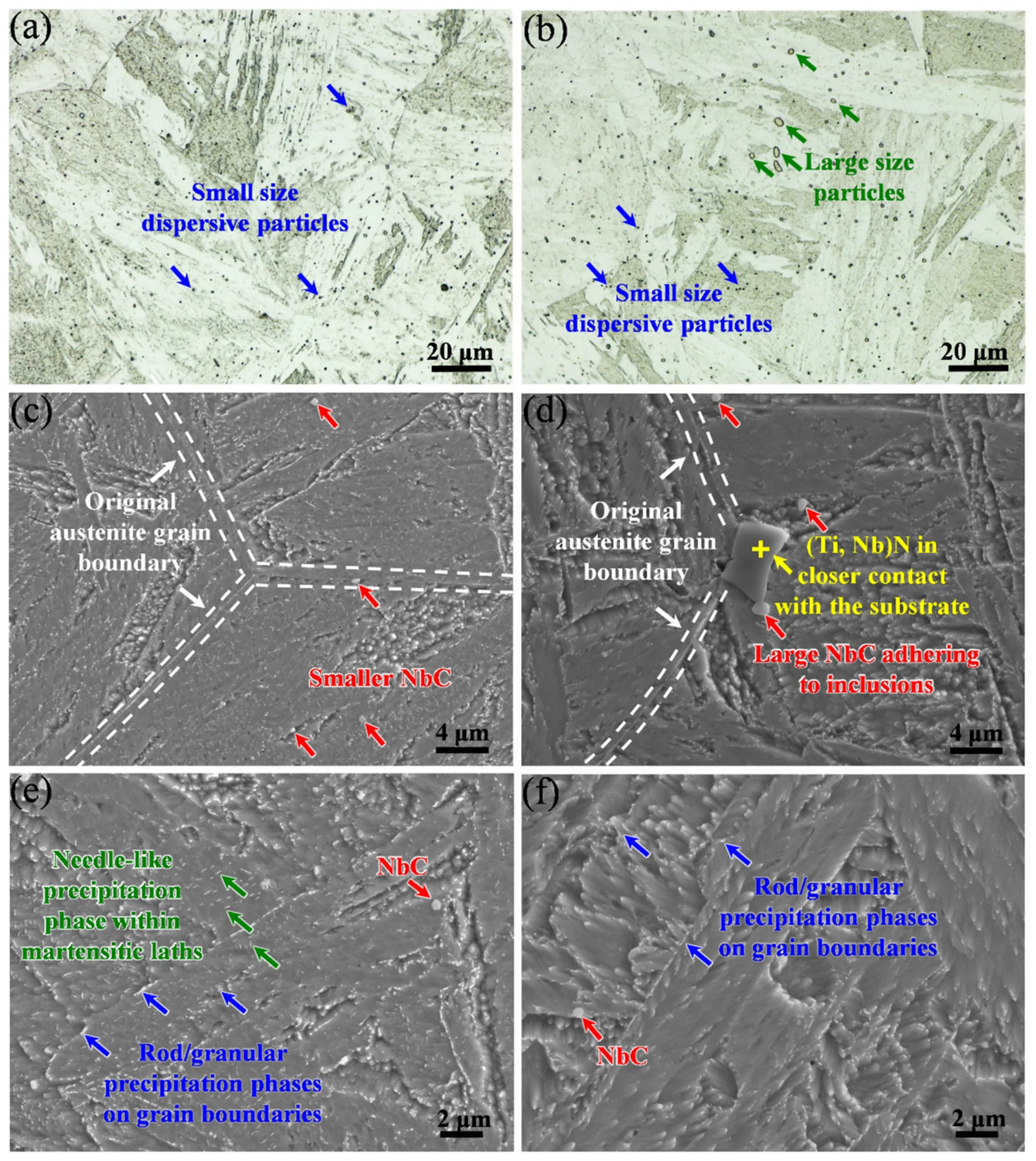
Microstructures of LTS and HTS after secondary tempering: (**a**) OM image of LTS; (**b**) OM image of HTS; (**c**,**e**) SEM images of LTS; (**d**,**f**) SEM images of HTS.

Figure 5 presents a systematic comparison of the microstructures of LTS and HTS after secondary tempering, with the phase identities in each micrograph verified by complementary EDS and TEM analyses reported elsewhere in this study. In LTS (Figure 5a,c,e), OM and SEM observations reveal smaller NbC particles distributed within grains (red arrows in Figure 5c,e), abundant needle-like precipitates within martensitic laths (green arrows in Figure 5e), and rod/granular precipitates along prior austenite grain boundaries (blue arrows in Figure 5e). The identification of these precipitates by high-resolution TEM imaging and selected-area diffraction will be presented in Figure 9. In HTS (Figure 5b,d,f), the microstructure differs markedly, with OM observation (Figure 5b) showing numerous coarse golden particles whose elemental composition, as determined by EDS point analysis and elemental mapping (Figure 2b, Figure 4), is consistent with that of (Ti,Nb)N inclusions, and which are absent in LTS. SEM further reveals that HTS contains almost no needle-like Cr_2_N precipitates within laths (Figure 5f), consistent with the reduced matrix N content consumed by (Ti,Nb)N formation, together with larger NbC particles, many of which are observed attached to (Ti,Nb)N inclusions (Figure 5d, yellow cross). This spatial association between NbC and (Ti,Nb)N supports the literature-reported heterogeneous nucleation mechanism whereby pre-formed TiN is expected to provide epitaxial nucleation sites for subsequent NbC precipitation, because TiN and NbC share the same NaCl-type crystal structure and close lattice parameters (aTiN ≈ 0.424 nm, aNbC ≈ 0.447 nm) that reduce the interfacial energy barrier [19]. Rod/granular M_23_C_6_ is also present on grain boundaries in HTS (blue arrows in Figure 5f), as verified by TEM (Figure 10b). The EDS elemental maps in Figure 4 and the quantitative EDS results in Table 2 further demonstrate that the matrix in HTS is depleted of Nb, N, Mo, and V relative to LTS, consistent with the formation of (Ti,Nb)N–NbC composite inclusions.

**Figure 6:**
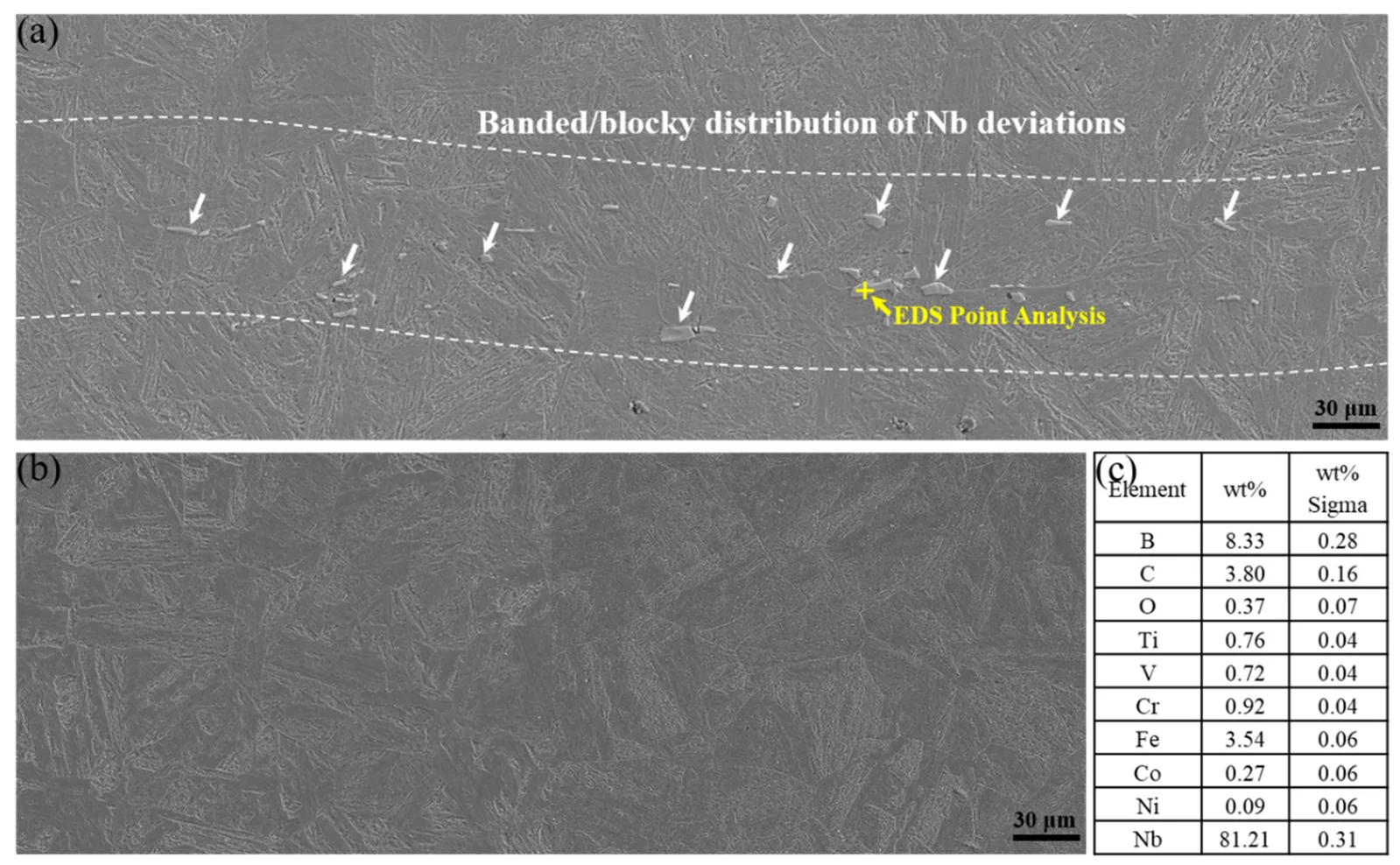
LTS/HTS Nb polarization SEM images: (**a**) HTS Nb polarization SEM images; (**b**) LTS SEM images; (**c**) quantitative results of band/block polarization EDS analysis.

Figure 6 shows SEM images of the microstructures of LTS and HTS. In HTS, numerous Nb segregations are observed (Figure 6a), with the corresponding EDS point analysis results shown in Figure 6c. Such Nb segregation consumes a substantial amount of Nb, thereby reducing its concentration in the matrix. In contrast, LTS exhibits almost no Nb segregation (Figure 6b), resulting in a comparatively higher Nb content within the matrix.

**Figure 7:**
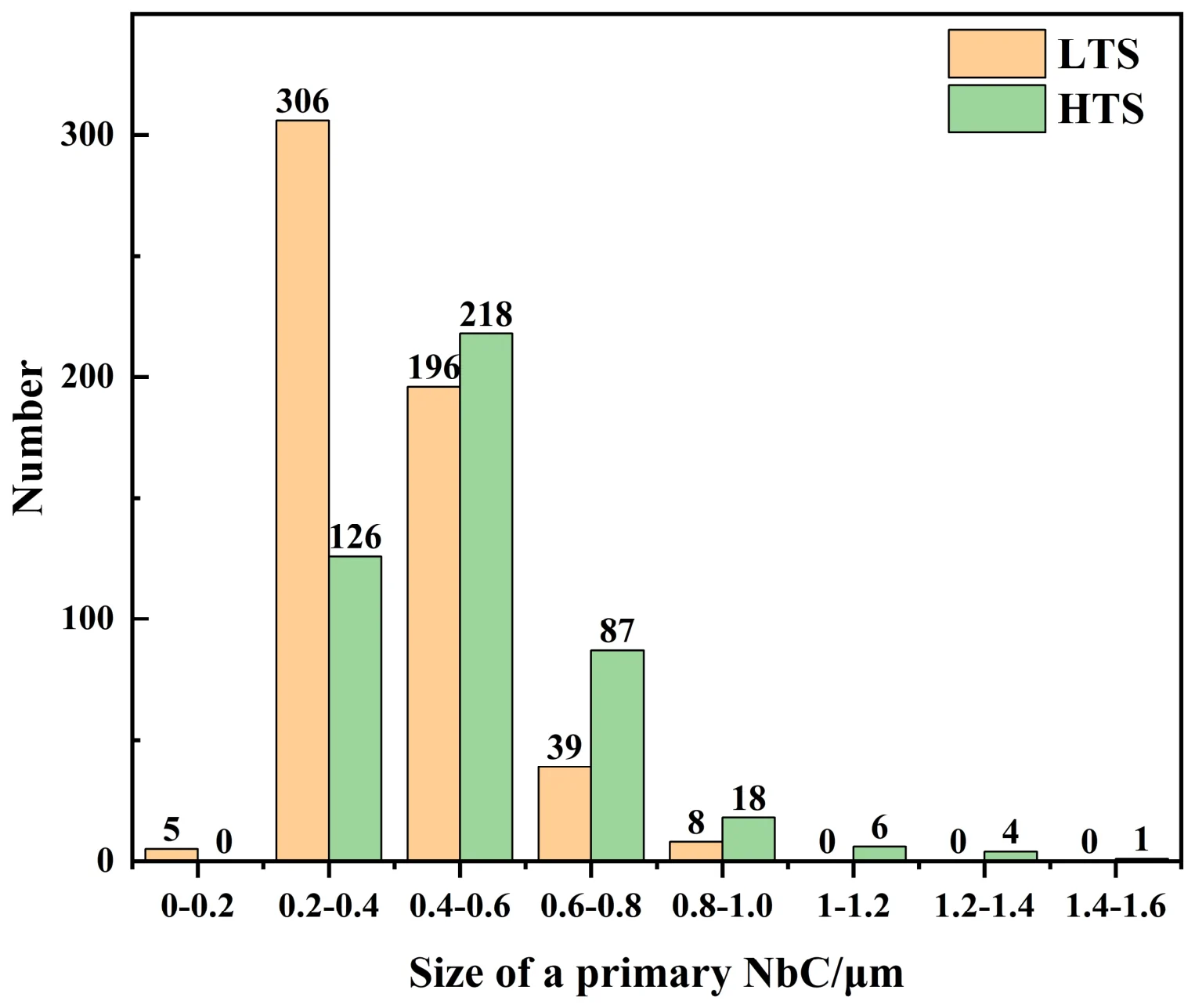
Size distribution statistics of the number of NbC in LTS/HTS.

Figure 7 shows the statistical analysis results of NbC in LTS and HTS. Compared with HTS, LTS contains a greater total number of NbC particles. In LTS, the highest frequency of NbC particles occurs in the 0.2–0.4 μm interval, with a notable contribution also present in the 0–0.2 μm range. In HTS, the maximum frequency is observed in the 0.4–0.6 μm interval, with a broader size distribution extending up to 1.6 μm. This indicates that LTS possesses finer and more uniformly dispersed NbC precipitates, whereas HTS exhibits coarser NbC particles with a wider size spread.

**Figure 8:**
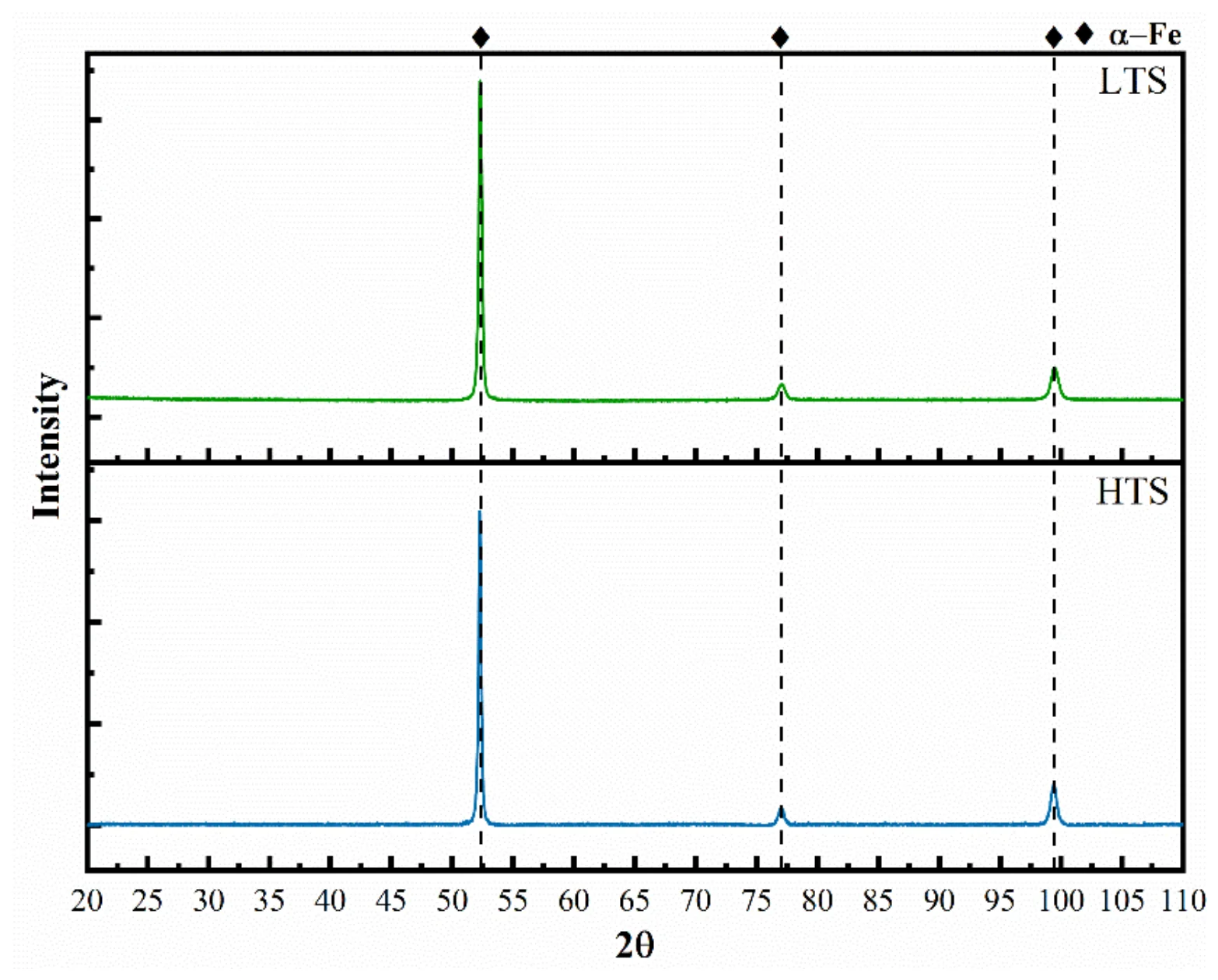
LTS/HTS secondary tempered state XRD physical phase identification.

**Figure 9:**
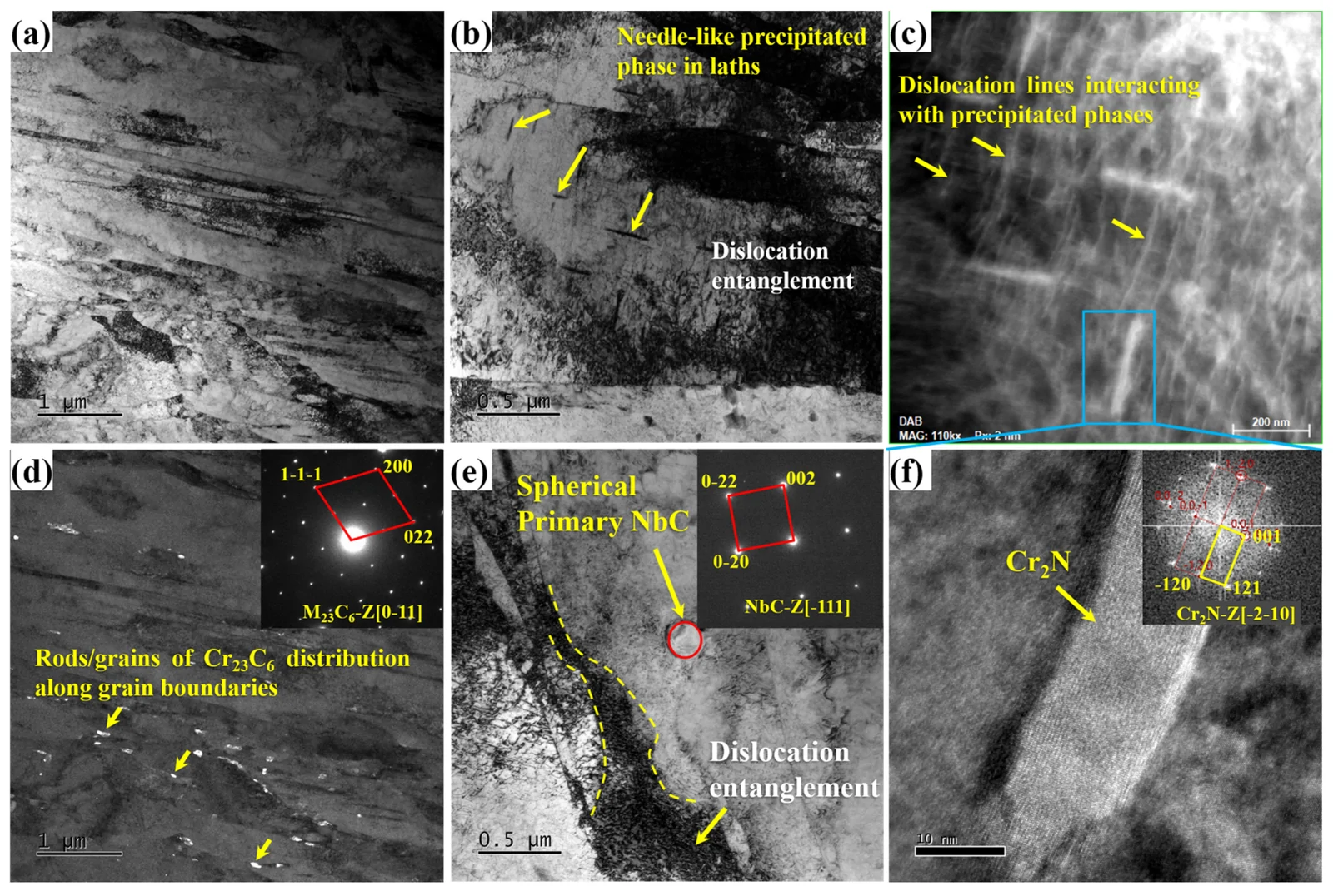
LTS TEM images: (**a**) bright-field (BF) image of martensitic laths; (**b**) needle-like precipitates and dislocation entanglement within laths; (**c**) high-angle annular dark-field scanning transmission electron microscopy (HAADF-STEM) image of interaction of needle-like precipitation phase and dislocation; (**d**) dark-field (DF) image of martensitic laths and Cr_23_C_6_ precipitates on lath boundaries, with the red circle indicating the region from which the selected area diffraction (SAD) pattern was acquired; (**e**) spherical NbC precipitates within laths (**f**) high-resolution image of needle-like precipitation phase, with the red square indicating the region from which the Fourier transform was acquired.

**Figure 10:**
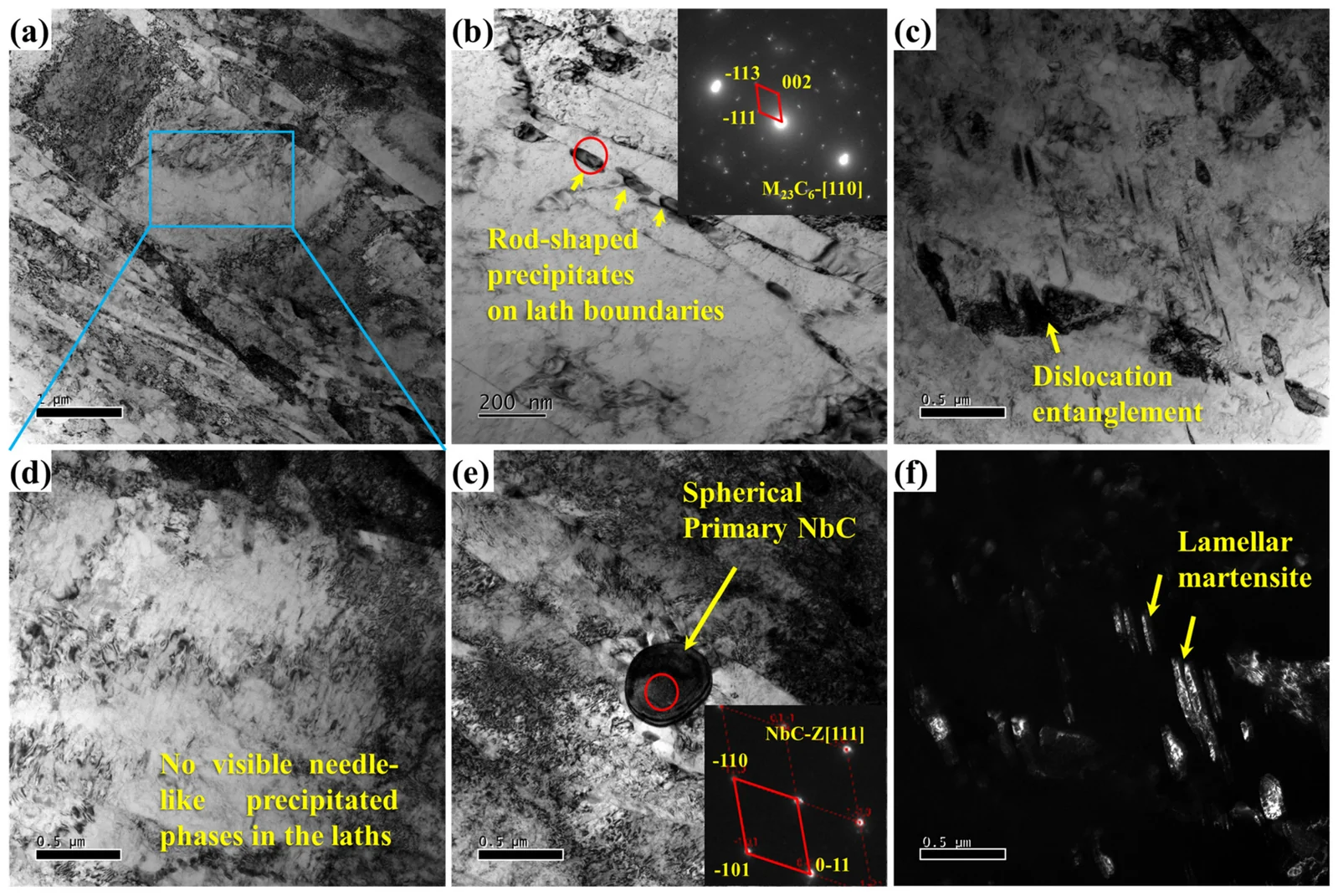
HTS TEM images: (**a**) martensitic lath image; (**b**) Cr_23_C_6_ precipitated phase on martensitic lath boundary; (**c**) lamellar martensitic BF image; (**d**) magnified image of martensitic lath; (**e**) spherical NbC precipitated phase; (**f**) lamellar martensitic DF image.

Figure 8 shows the XRD patterns for phase identification in LTS and HTS after secondary tempering. The diffraction peaks are indexed as α-Fe, which is consistent with the body-centered cubic structure of tempered martensite. Although the lattice parameters of BCC ferrite and martensite are indeed similar, the microstructural characterization by TEM (Figure 9a and Figure 10a) confirms that the matrix consists of lath martensite with a high dislocation density, rather than equiaxed ferrite. No austenite (FCC peaks were detected in either sample, indicating complete martensitic transformation following quenching.

Figure 9 shows TEM images of the microstructure and precipitates in the LTS. As shown in Figure 9a, the LTS sample exhibits pure martensitic laths, within which needle-like precipitates are observed (Figure 9b) interacting with dislocation lines (Figure 9c). Granular Cr_23_C_6_ precipitates on the martensitic lath boundaries in LTS (Figure 9d), whereas primary NbC precipitates and dislocation tangles in the laths (Figure 9e). The high-resolution TEM image in Figure 9f reveals that the needle-like precipitates within the martensitic laths of the LTS are Cr_2_N phases.

Figure 10 shows TEM images of the microstructures and precipitates in the HTS. As shown in Figure 10a, the HTS sample exhibits pure martensitic laths, and rod-shaped Cr_23_C_6_ precipitates are observed along the martensitic lath boundaries as shown in Figure 10b. In addition, numerous lamellar martensite structures intersect with dislocations, forming dislocation entanglements, as shown in Figure 10c. It is worth noting that no obvious needle-like precipitates are observed within the laths in HTS, which differs from the microstructure in LTS. Furthermore, HTS contains primary NbC precipitates and dislocation tangles within the laths, with the NbC size in HTS being larger than that in LTS, consistent with previous results.

**Figure 11:**
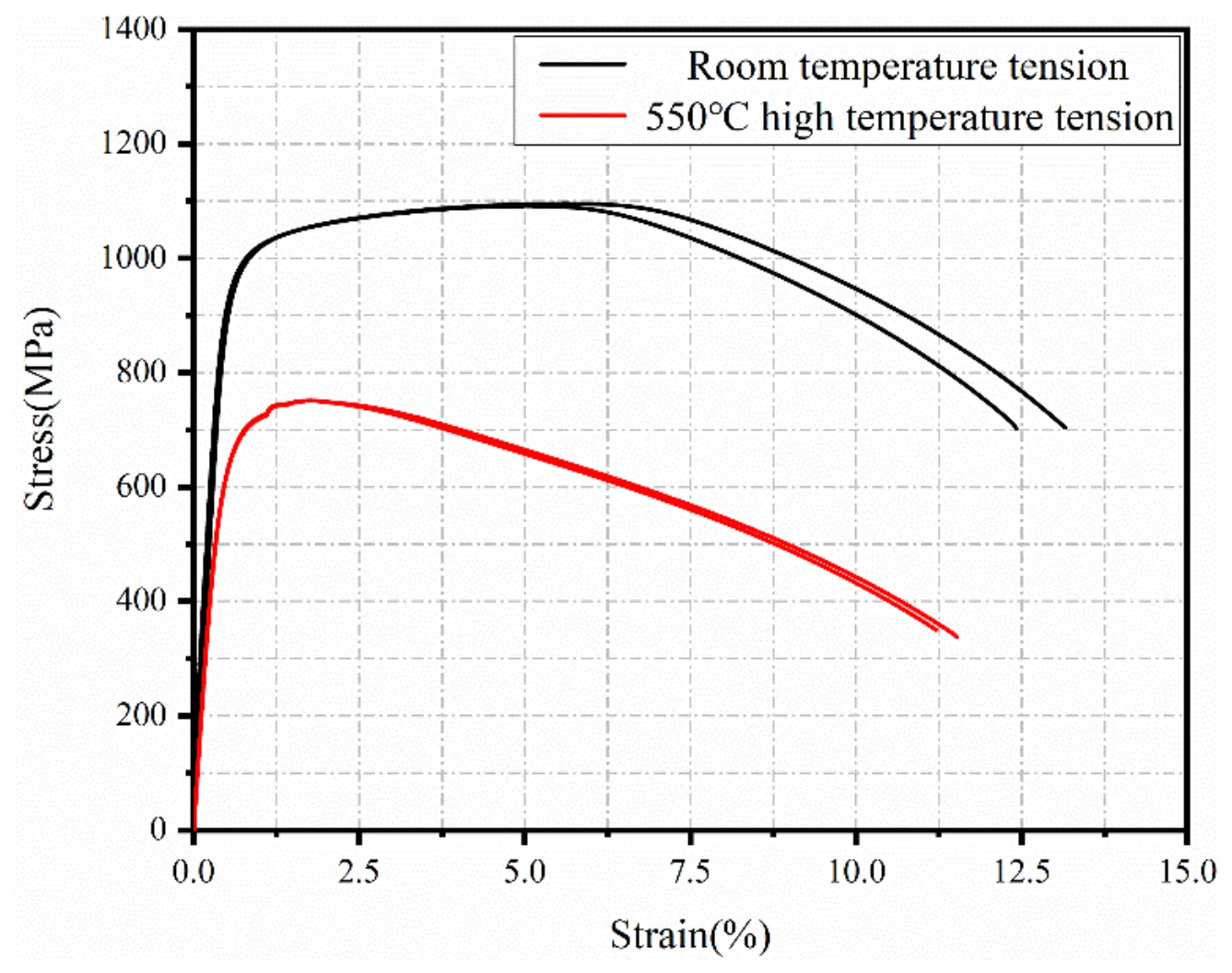
HTS room-temperature/550 °C high-temperature uniaxial tensile properties curve.

Figure 11 shows the room-temperature and 550 °C tensile curves of HTS, and the corresponding mechanical properties are summarized in Table 3 and Table 4, respectively. At room temperature, HTS exhibits a tensile strength (Rm)of 1094 MPa, a yield strength (Rp0.2) of 962.5 MPa, an elongation after fracture (A) of 16.25%, and a reduction in area (Z) of 61.5%. These values fall within the range reported for Jethete M152 (a comparable Cr–Mo–V–Nb–N martensitic stainless steel in the hardened and double-tempered condition), for which the AMS 5719 and BS S151 specifications give a tensile strength of 950–1150 MPa, a yield strength of 800–900 MPa, and an elongation of 12–14% [4]. The present HTS matches the tensile strength range of Jethete M152 while exhibiting a higher yield strength and superior ductility. At 550 °C, HTS shows a tensile strength of 751.3 MPa and a yield strength of 653 MPa. Systematic elevated temperature tensile data for Jethete M152 are scarce in the open literature; therefore, the 550 °C properties are compared with the Chinese aviation standard WS 9-6521-78 specified for 1Cr10Co6MoVNbN steel (tensile strength ≥ 830 MPa; yield strength ≥ 740 MPa at 550 °C). The HTS values fall below the standard limits by 78.7 MPa (≈9.5%) and 87 MPa (≈11.8%), respectively. The elevated temperature elongation (22%) and reduction in area (73.81%) remain above the standard values (19% and 70%, respectively). This high-temperature strength deficit is discussed in Section 4 in terms of the microstructural degradation associated with excessive (Ti,Nb)N formation.

**Figure 12:**
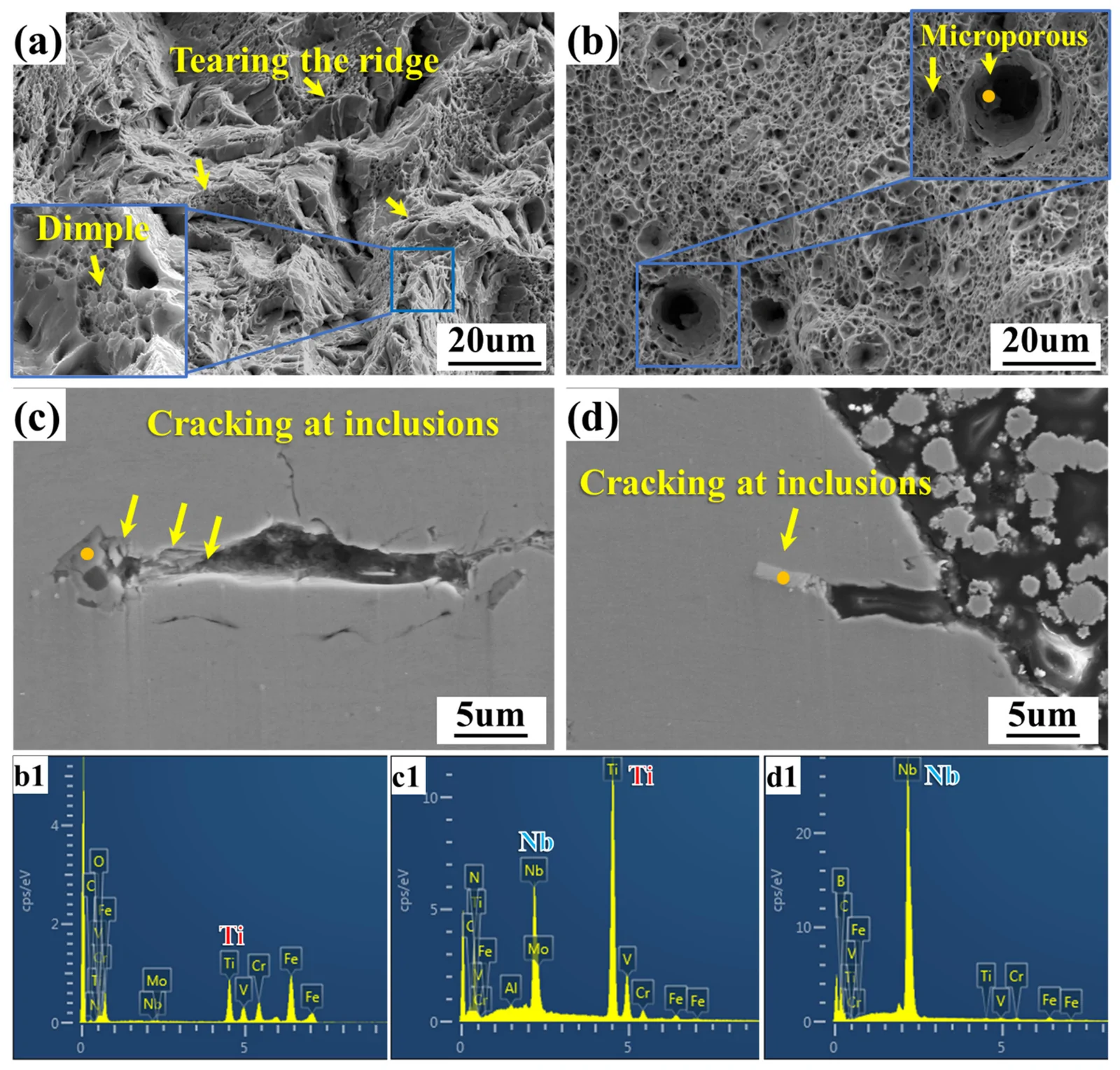
SEM images of HTS room-temperature/550 °C high-temperature tensile fractures: (**a**) room-temperature tensile fracture; (**b**) high-temperature tensile fracture, with (**b1**) showing the EDS point analysis of point b1; and (**c**,**d**) high-temperature tensile crack sprouting sources, with (**c1**) and (**d1**) showing the EDS point analyses of points c1 and d1, respectively.

Figure 12 shows the fracture morphologies of HTS after tensile testing at room temperature and at 550 °C. At room temperature (Figure 12a), the fracture surface exhibits a mixed ductile–brittle morphology, characterized by a combination of dimples and tearing ridges. This indicates that the specimen underwent partial plastic deformation but retained a tendency for brittle fracture under the applied stress. At 550 °C (Figure 12b), the fracture surface is dominated by dimples, with no evident cleavage facets or tearing ridges, indicating a fully ductile fracture mode. Large pores containing angular square-shaped particles are observed on the 550 °C fracture surface (Figure 12c,d); EDS analysis identifies these particles as (Ti,Nb)N inclusions. Examination of the fracture cross-section further reveals that crack initiation is associated with (Ti,Nb)N inclusions and regions of macroscopic Nb segregation, which act as stress concentrators under high-temperature loading. The incompatibility in plastic deformation between the hard angular inclusions and the surrounding matrix promotes local stress concentration, leading to void nucleation, growth, and eventual coalescence into the primary crack.

**Figure 13:**
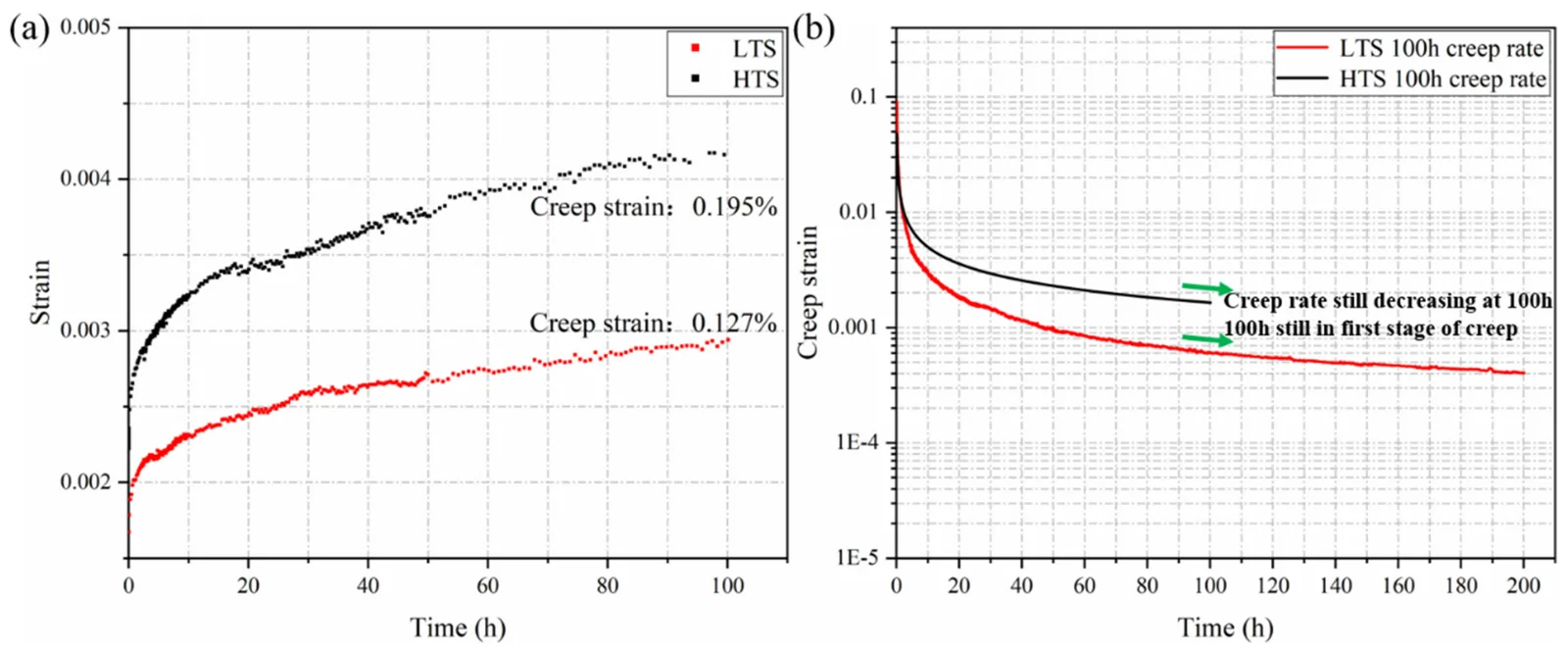
LTS/HTS 100 h short-term creep curves: (**a**) time–strain curve; (**b**) time–creep rate curve.

Figure 13 shows the short-term creep curve of LTS and HTS. As shown in Figure 13a, the creep strain of LTS after 100 h is 0.127%, whereas that of HTS is 0.195%, representing an increase of approximately 53% in HTS relative to LTS. From the creep strain rate curve in Figure 13b, it is observed that the creep rate continuously decreases within 0–200 h. This indicates that, at the end of 100 h creep, the material remains in the primary stage of creep, where the total dislocation multiplication rate exceeds the dislocation recovery rate. The creep rate of HTS is consistently higher than that of LTS within 100 h, suggesting a higher density of mobile dislocations and a faster dislocation movement rate [20,21]. These differences are attributed to the heat treatment condition and the combined effects of dislocation–precipitate interactions and matrix solid solution strengthening, respectively.

**Figure 14:**
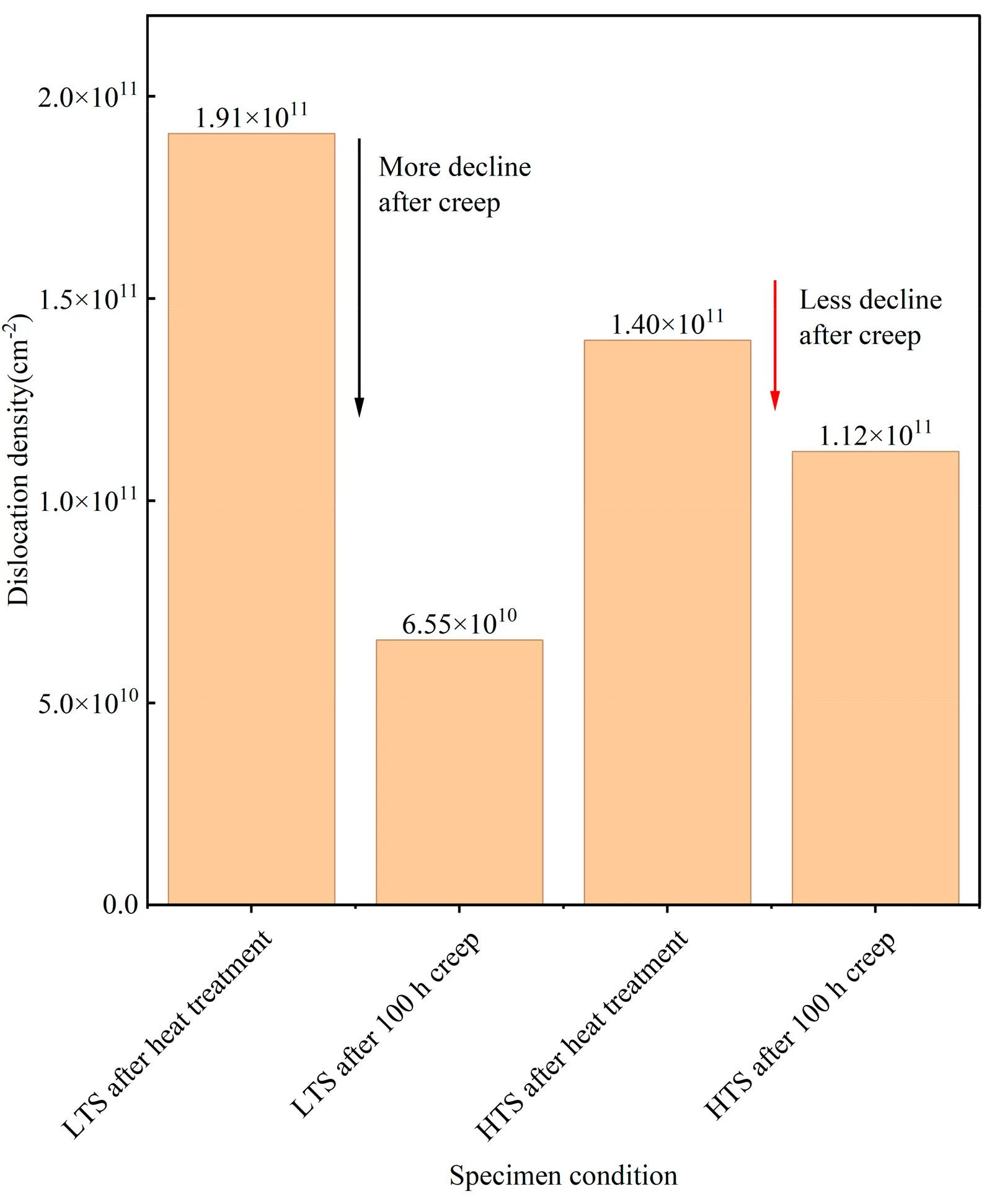
Mobile dislocation density of LTS and HTS before and after 100 h creep, determined by XRD line profile analysis using the modified Williamson–Hall method. The y-axis represents mobile dislocation density (×10^14^ m^−2^).

Figure 14 shows dislocation densities in LTS and HTS before and after 100 h creep, as determined from XRD measurements. According to the theory in the literature [22,23], the dislocation density measured by XRD predominantly corresponds the density of mobile dislocation. As shown in Figure 14, the mobile dislocation densities of HTS and LTS before creep are of the same order of magnitude and essentially comparable. After 100 h of creep, however, the mobile dislocation density of LTS decreases remarkably, whereas that of HTS decrease is not significant. This suggests that during the creep process of LTS, the motion of mobile dislocations is more strongly impeded by precipitates or lath boundaries, resulting in a rapid reduction in their density. In contrast, the slower decreases in mobile dislocation density for HTS indicates weaker hindrance, which contributes to its larger creep strain.

It should be noted that the dislocation density measured by XRD line profile analysis represents an effective or average value, and the distinction between mobile and immobile dislocations is inferred from the comparative analysis of creep behavior rather than directly resolved by the XRD method.

**Figure 15:**
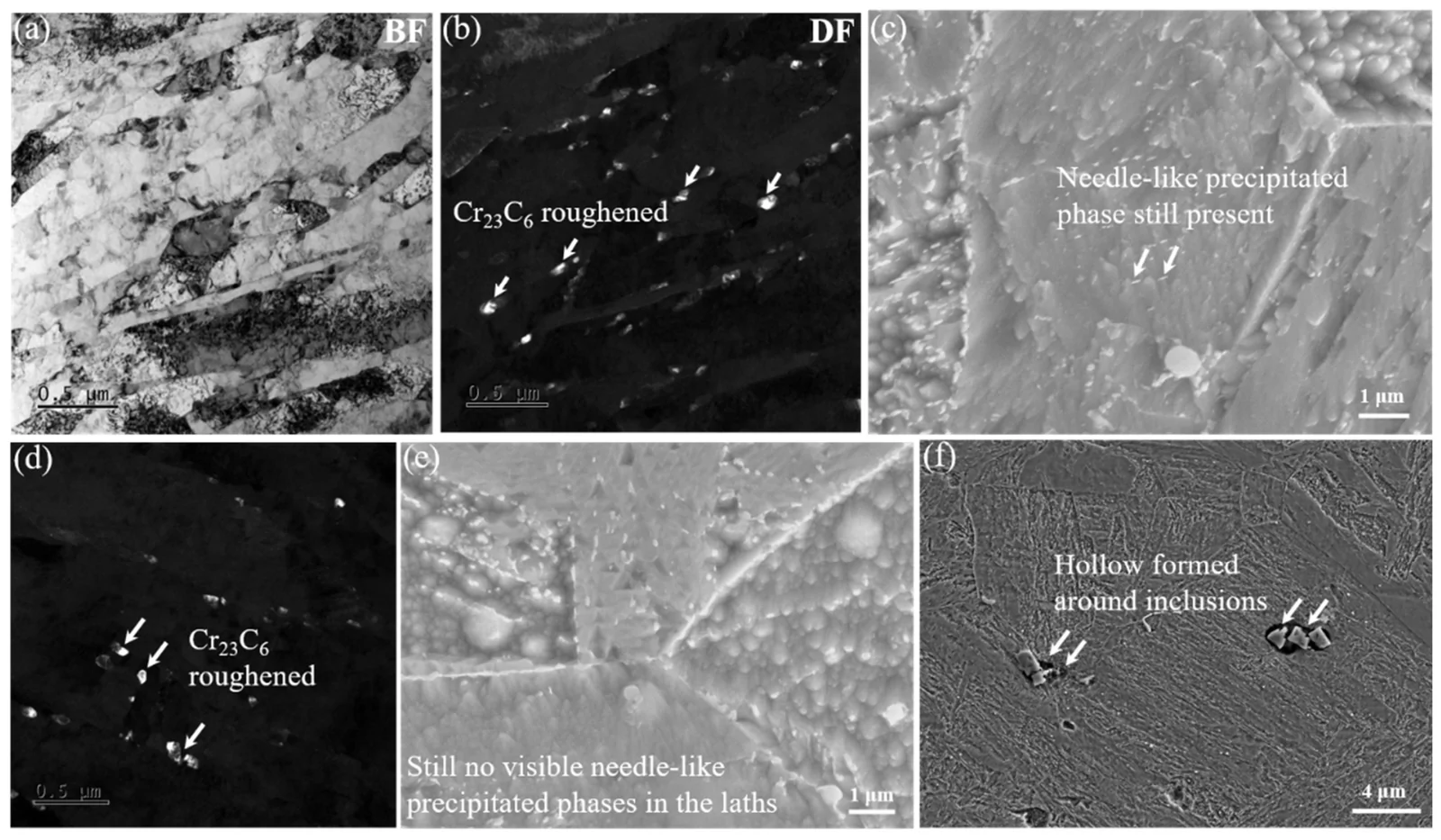
Morphology of LTS and HTS after 100 h short-term creep: (**a**) TEM-BF image of martensitic laths and Cr_23_C_6_ of LTS, (**b**) TEM-DF image of martensitic laths and Cr_23_C_6_ of LTS, (**c**) SEM image of LTS, (**d**) TEM-DF image of martensitic laths and Cr_23_C_6_ of HTS, (**e**) SEM image of HTS, and (**f**) formation of holes around inclusions in HTS.

Figure 15 shows the microstructure morphologies of LTS and HTS after creep observed by TEM and SEM, where Figure 15a–c illustrate the microstructure morphologies of LTS, while Figure 15d–f illustrate the microstructure morphologies of HTS. As can be seen from Figure 15b,d, the M_23_C_6_ phases in LTS/HTS have a certain degree of coarsening compared with those before creep. Figure 15c shows that M_2_X phase in LTS still exhibits a higher stability, and does not coarsen after 100 h creep, while in HTS there is still no such M_2_X phase (Figure 15e). As can be seen from Figure 15f, there are some obvious changes in the morphology around the inclusions after creep in HTS. It was found that the inclusions and the matrix were tightly connected without obvious gaps before 100h creep (as shown in Figure 5d); after creep, some pores appear between the inclusions and the matrix, which is believed to be caused by the stress concentration between the inclusions and the matrix during creep.

## 4. Discussion

This study investigated the microstructure, inclusions, and precipitates of 1Cr10Co6MoVNbN steels with varying Ti contents, and evaluated their tensile properties and short-term creep performance. As shown in Figure 2 and Figure 3, the number of (Ti,Nb)N inclusions in the high-Ti steel were significantly higher than that in the low-Ti steel.

**Figure 16:**
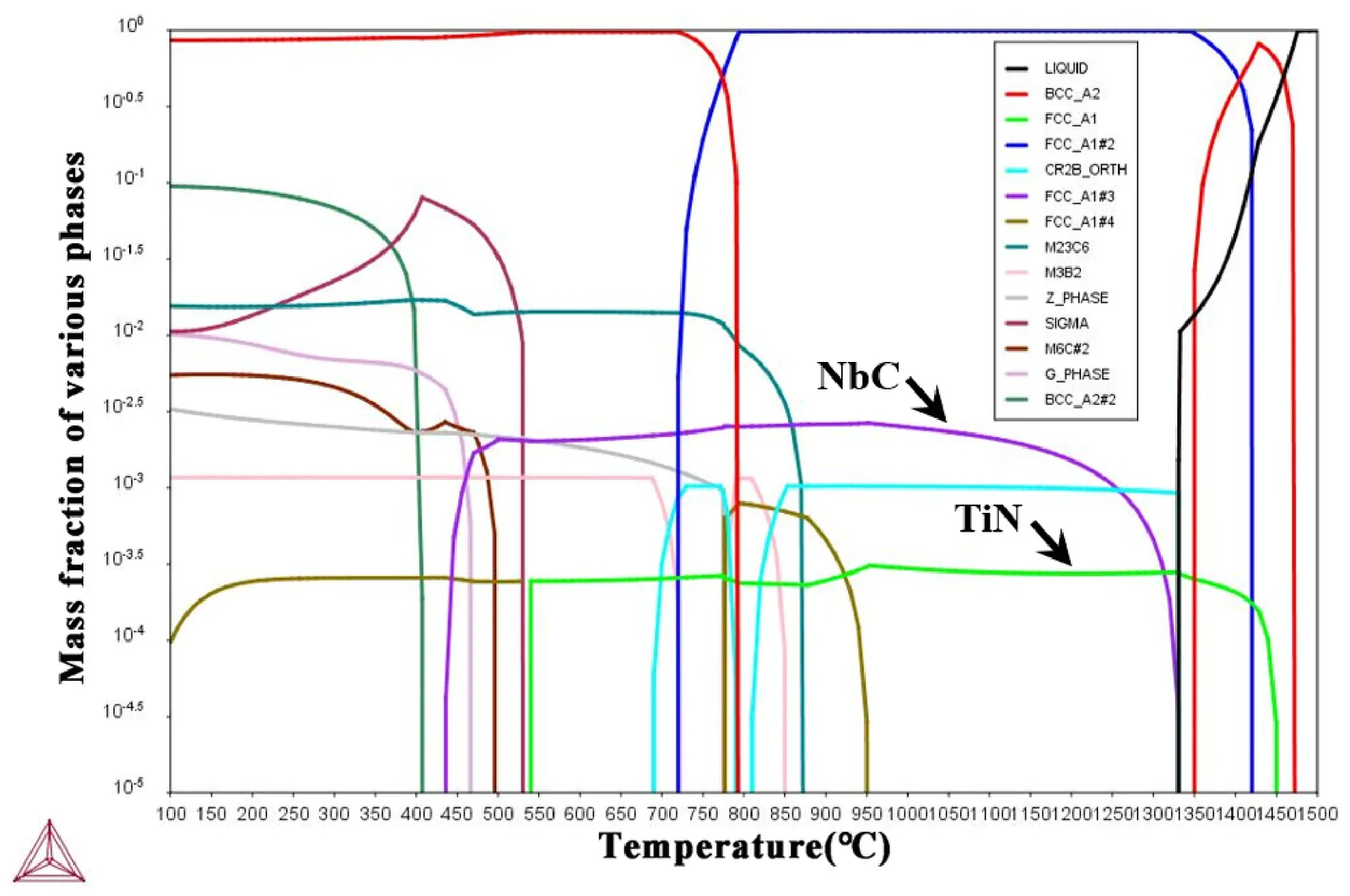
HTS equilibrium phase diagram simulation (Thermal Calc^®^ 2015b).

Because the TiN and NbC have higher melting than other compounds, they precipitated first during solidification of the molten steel. As shown in Figure 16, thermodynamic simulations of the HTS indicated that TiN formed initially, followed by primary NbC. Owing to their similar crystal structure and lattice parameters, these precipitates combined to form a mixed nitride phase, (Ti,Nb)N, which was further confirmed by SEM observations.

The formation of a large number of (Ti, Nb)N inclusions consumed more N element from the matrix, thereby reducing the formation of Cr_2_N precipitates, which require N participation. This trend is consistent with previous studies on the optimal N:Al ratio in martensitic steels [6,7], where excessive Al promotes the preferential formation of AlN, consequently lowering the N available for MX carbonitride formation. An insufficient MX number density weakens the stabilization of the substructure, leading to reduced creep strength. Similarly, in the high-Ti steel (HTS), the relatively high Ti content resulted in the early formation of TiN during solidification, as predicted by the phase diagram simulation in Figure 16. These TiN particles provided nucleation sites for Nb, which readily segregates, leading to the precipitation of coarse spherical NbC and the development of Nb-rich of strip-like segregations (Figure 4, Figure 5 and Figure 6). Due to the similar crystal structure and lattice constant of TiN and NbC, composite inclusions of (Ti,Nb)N formed, trapping strengthening elements such as Nb, N, V, Mo in the matrix and producing (Ti,Nb)N and coarse NbC with limited contribution to strengthening. As shown in Figure 7, LTS did not form significant TiN, allowing for NbC to nucleate and precipitate more uniformly, producing finer NbC particles capable of providing dispersion strengthening. The addition of N also promotes the transformation of Cr_7_C_3_ into the stable M_2_X phase, enhancing secondary hardening [14]. However, in HTS, the consumption of N by (Ti,Nb)N reduced M_2_X precipitation in the matrix. Since most M_2_X phases precipitated intragranularly, where they could hinder dislocation motion, the higher N content retained in LTS promoted greater precipitation of needle-like M_2_X within grains (Figure 9f), effectively obstructing dislocation movement and climb, thereby improving creep strength (Figure 9c). Consequently, the size distribution of NbC and M_2_X phase played a critical role in determining mechanical properties of this steel. The finer NbC and abundant needle-like Cr_2_N in LTS effectively hindered dislocation motion and climb, whereas the coarser NbC and absence of Cr_2_N in HTS led to weaker resistance to dislocation movement. These microstructural differences directly account for the substantially higher creep strain observed in HTS (0.195%) compared with LTS (0.127%) after 100 h at 550 °C (Figure 13).

**Figure 17:**
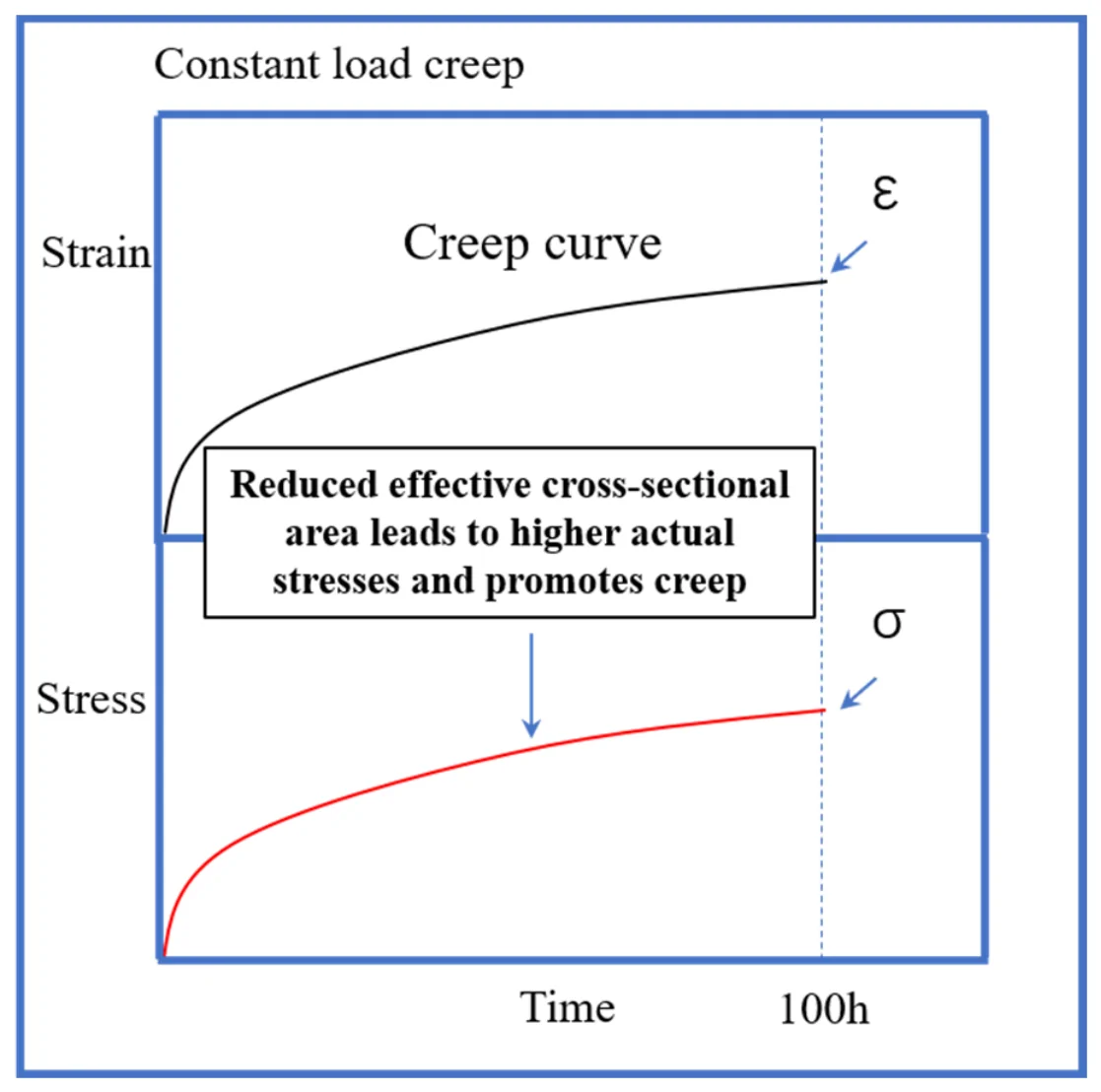
Creep voids lead to an increase in actual creep stress.

To elucidate the relative influence of the identified microstructural changes on creep resistance, the total creep strength σ_creep_ can be expressed schematically as the sum of several strengthening contributions:

σ_creep_≈σ_0_ + Δσ_ss_ + Δσ_ppt_ + Δσ_disl_

where σ_0_ is the intrinsic matrix strength, Δσ_ss_ is the solid solution strengthening contribution, Δσ_ppt_ is the precipitation strengthening contribution, and Δσ_disl_ is the dislocation strengthening contribution. The Δσ_ss_ scales with the concentration of substitutional and interstitial solutes in the matrix. EDS analysis (Table 2) shows that the HTS matrix is depleted of Nb, Mo, V, and N relative to LTS. A lower solute content reduces Δσ_ss_, thereby lowering the resistance to dislocation glide. However, because the absolute depletion is modest (e.g., Nb drops from ~0.33 at. % to ~0.22 at. %), the contribution of this term to the overall creep acceleration is expected to be secondary. The precipitation strengthening contribution (Δσ_ppt_) follows the Orowan mechanism, scaling with the average precipitate size (d) and the inter-precipitate spacing (λ) as Δσ_ppt_ ∝ d/λ, where d represents the average precipitate diameter and λ denotes the mean inter-precipitate spacing. In LTS, the presence of abundant fine Cr_2_N needles (d ~ 50–200 nm, Figure 9f) and uniformly dispersed NbC particles (d < 400 nm, Figure 7) provides effective barriers to dislocation motion. In HTS, the near-absence of Cr_2_N (Figure 5f, Figure 10) and the coarsening of NbC (majority 0.4–1.6 μm, Figure 7) cause a marked reduction in Δσ_ppt_. Because Cr_2_N precipitates intragranularly and directly obstructs dislocation climb, a dominant deformation mode during primary creep at 550 °C, the loss of this phase appears to be the most significant contributor to the accelerated creep in HTS. The XRD results (Figure 14) indicate that LTS exhibits a rapid decrease in mobile dislocation density during creep, consistent with strong dislocation pinning by fine precipitates. In HTS, the slower decline in mobile dislocation density reflects weaker pinning, allowing for continued dislocation motion and strain accumulation. TEM-based line intersection counting was not performed in this study owing to its limited statistical representativeness and the difficulty of distinguishing mobile from immobile dislocations in complex tempered martensitic microstructures. However, the TEM observations (Figure 9 and Figure 10) show stronger dislocation pinning by fine precipitates in LTS and weaker pinning in HTS, which is consistent with the XRD-derived mobile dislocation density trends. Beyond the intrinsic strengthening terms, the nucleation and growth of voids around (Ti,Nb)N inclusions (Figure 15f, Figure 17) reduce the effective cross-sectional area A_eff_. The resulting increase in true stress (σ_true_ = F/A_eff_) provides an additional acceleration mechanism that is absent in LTS. Based on this qualitative assessment, the loss of fine Cr_2_N precipitation appears to be the dominant factor in the elevated temperature strength deficit of HTS, because it removes the primary intragranular barrier to dislocation climb during creep. The coarsening of NbC and the modest solid solute depletion act as secondary contributors, while void-induced stress amplification becomes progressively more important as creep strain accumulates.

The microstructural degradation of HTS described above also explains the discrepancy between its qualified room-temperature tensile properties and its unqualified high-temperature tensile properties (Figure 11). At 550 °C, the depletion of solid solution-strengthening elements (Nb, Mo, V) and the reduction of fine Cr_2_N precipitation in HTS lower the matrix resistance to plastic deformation, making the material more susceptible to inclusion-initiated void nucleation. The pronounced susceptibility of metallic materials to inclusion-initiated void nucleation and crack propagation during high-temperature tensile deformation—contrasting with their relative insensitivity at room temperature—is attributed to fundamental changes in deformation mechanisms and thermal softening, and environmental interactions. At room temperatures, plastic deformation was dominated by dislocation slip. Stress concentrations near inclusions were often accommodated by matrix plasticity through dislocation multiplication and rearrangement, thereby limiting void nucleation primarily to large inclusions [24]. These microvoids exhibit limited growth due to dislocation pile-ups and localized strain hardening, and they rarely coalesce into critical cracks before macroscopic failure [25]. In contrast, elevated temperatures activate three synergistic degradation pathways: (1) thermal softening markedly reduces the matrix yield strength, thereby diminishing resistance to interfacial decohesion at inclusion/matrix boundaries [26]; (2) diffusion-assisted mechanisms, including vacancy migration driven by stress gradients and accelerated atomic diffusion, promote void nucleation at inclusion interfaces and enable rapid void growth via vacancy absorption (diffusion-controlled growth) [27]; and (3) grain boundary sliding in polycrystalline alloys induces severe stress concentrations at inclusions located on grain boundaries or triple junctions [28]. Under sustained high-temperature loading, these processes act synergistically with creep damage accumulation, whereby inclusions serve as preferential sites for void clustering and microcrack linkage. This phenomenon has been validated in Grade 91 steel, where MnS inclusions accelerate creep void formation compared to inclusion-free regions [29,30]. Consequently, the fracture mechanism at high-temperature transitions from inclusion-independent modes (e.g., crystallographic slip) to inclusion-dominated failure, characterized by fracture surfaces with inclusions at the voids centers and a marked reduction in ductility.

The inferior creep performance of HTS is further corroborated by the XRD dislocation density analysis (Figure 14), which shows that the mobile dislocation density in LTS decreased rapidly during creep due to enhanced obstacles to dislocation motion, whereas HTS exhibited a much slower decline. This difference reflects the stronger impediment to dislocation motion in LTS provided by its finer precipitate distribution. Moreover, the results in Figure 10 and Figure 15 showed that the M_2_X phase in LTS remained stable before and after creep, whereas no such stability was observed in HTS. Due to the higher N content in LTS matrix compared with the HTS matrix, LTS precipitated a greater number of needle-like M_2_X phases within grains and martensitic laths, which strongly hindered dislocation movement and climb, thereby improving the creep strength.

In addition to the precipitate effects, incompatible plastic deformation between the inclusions and the matrix under high-temperature loading resulted in local stress concentration (Figure 15f). The (Ti,Nb)N inclusions act as local stress concentrators that promote void nucleation at the inclusion–matrix interface during creep (Figure 15f). Given the relatively high number density of these inclusions in HTS, the inter-particle spacing is comparable to the inclusion size, raising the possibility that neighboring voids may eventually grow and coalesce to form a continuous crack. However, the present post-creep micrographs do not capture the transient coalescence stage directly; therefore, the involvement of (Ti,Nb)N inclusions in void linkage remains a plausible inference rather than a confirmed mechanism. Regardless of the exact linkage pathway, the development of voids reduces the effective cross-sectional area of the specimen, consequently increasing its true creep stress, as shown in Figure 17. Such an increase in true creep stress induced greater creep strain, thereby reducing the creep strength and creep life. Consequently, HTS exhibited a higher true creep stress than LTS due to the large number of inclusion-induced voids formed during creep, which also contributed to a greater creep strain. In summary, (Ti,Nb)N promoted short-term creep deformation in HTS through two mechanisms: (1) by depleting the matrix of strengthening elements and altering the size and distribution of precipitates, and (2) by generating voids during creep, which increased the true creep stress and consequently accelerated creep deformation.

**Figure 18:**
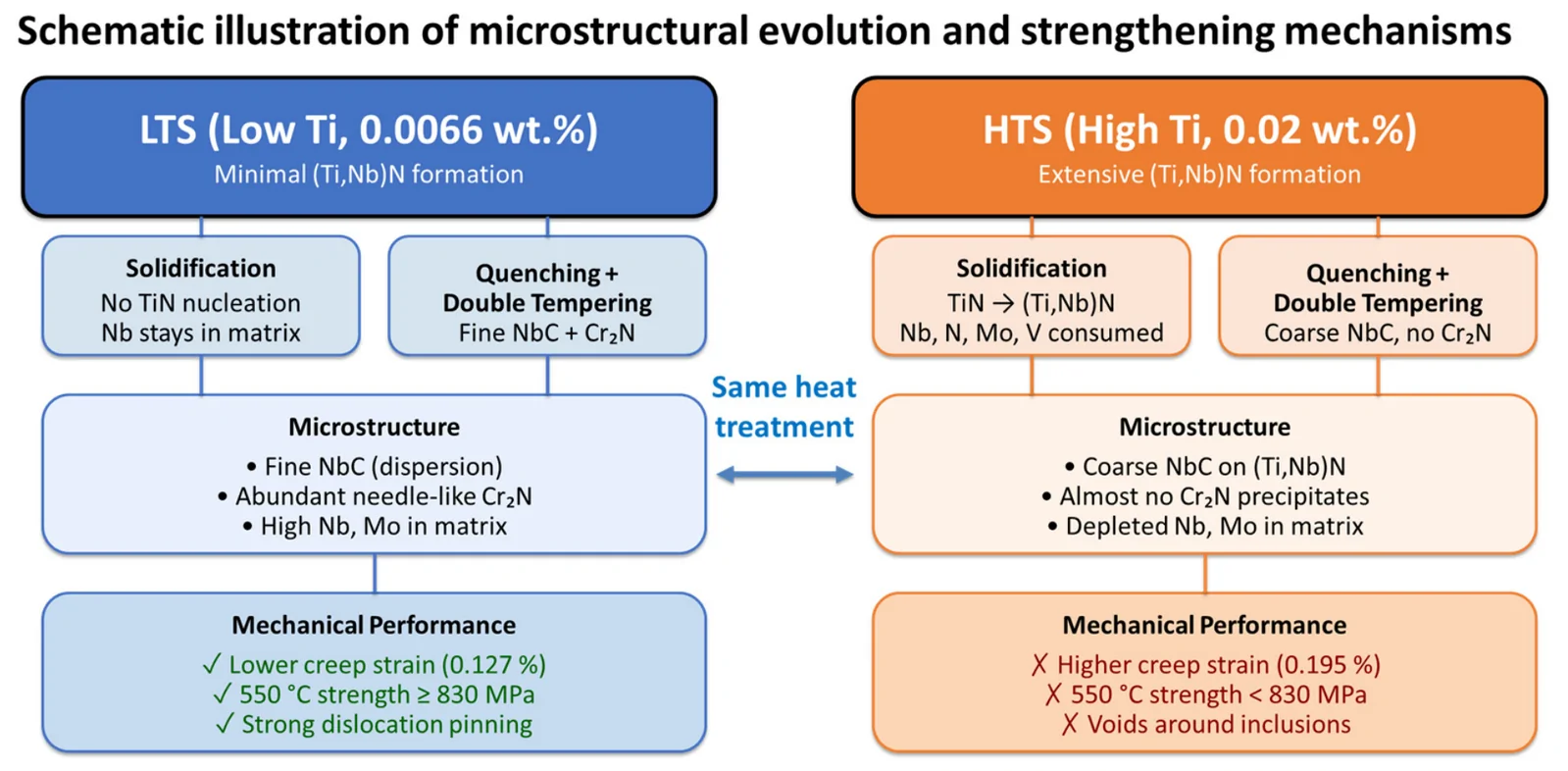
Schematic illustration of the effect of Ti content on microstructural evolution and mechanical properties in 1Cr10Co6MoVNbN steel.

To summarize the complex interplay between Ti content, microstructural evolution, and mechanical performance described above, a schematic illustration is presented in Figure 18. This figure juxtaposes the entire process chain for LTS and HTS—from solidification through heat treatment to final mechanical behavior—and highlights how the identical heat treatment parameters applied to both alloys produce fundamentally different outcomes depending on the Ti content. The schematic explicitly identifies the loss of fine Cr_2_N precipitation and the coarsening of NbC as the primary microstructural factors responsible for the accelerated creep and reduced high-temperature tensile strength in HTS, while also emphasizing the secondary contribution of void formation around (Ti,Nb)N inclusions. We believe this illustration provides readers with a clear, integrated overview of the degradation mechanisms identified in this study.

## 5. Conclusions

The effects of varying Ti contents on the tensile properties and short-term creep performance of 1Cr10Co6MoVNbN steel were investigated. OM, SEM, TEM, and XRD were employed to characterize its microstructure. The results of mechanical tests and microstructure analyses were summarized as follows:

1. The Ti content significantly influenced the amount of the (Ti,Nb)N phase. HTS contained a substantially higher number density of (Ti,Nb)N inclusions than LTS. EDS analysis indicated that the matrix of HTS was depleted of Nb, N, Mo, and V relative to LTS, consistent with the preferential formation of (Ti,Nb)N during solidification. Two characteristic types of (Ti,Nb)N composite inclusions were identified, including TiN core (Ti,Nb)N with surrounding primary NbC, and alumina core (Ti,Nb)N with attached primary NbC.

2. The Ti content also affected the content of the M_2_X phase, leading to differences in creep performance. LTS contained more needle-like Cr_2_N precipitates within grains and martensitic laths. In contrast, HTS exhibited fewer Cr_2_N precipitates and weaker Cr_2_N strengthening because the formation of abundant (Ti,Nb)N reduced the N content in the matrix, leading to a decrease in Cr_2_N precipitation. Consequently, HTS exhibited a larger creep strain.

3. The formation of numerous angular inclusions induced local stress concentrations during creep, leading to voids formation around them. The development of voids reduced the effective cross-sectional area, thereby accelerating the accumulation of creep strain.

## Figures and Tables

**Table 1:** Chemical compositions of 1Cr10 materials with different Ti contents (wt. %).

Materials	Fe	C	Mn	Cr	Mo	V	Nb	Ti	N	Co
LTS	Balance	0.089	0.64	10.32	0.82	0.23	0.21	0.0066	0.02	5.97
HTS	Balance	0.094	0.91	10.70	0.81	0.24	0.22	0.02	0.024	5.83

**Table 2:** EDS quantitative analysis results of LTS/HTS.

LTS					HTS			
Region	Ti	Nb	Mo	V	Ti	Nb	Mo	V
1	0.00	0.35	1.00	0.22	0.00	0.09	0.24	0.1
2	0.03	0.25	0.74	0.22	0.02	0.03	0.32	0.1
3	0.06	0.22	0.85	0.17	0.03	0.50	0.55	0.35
4	0.00	0.26	0.92	0.18	0.00	0.08	0.65	0.24
5	0.00	0.38	1.04	0.30	0.00	0.15	0.72	0.22
6	0.03	0.25	0.74	0.30	0.06	0.44	0.93	0.32
7	0.07	0.58	1.15	0.16	0.11	0.04	0.97	0.12
8	0.03	0.5	1.07	0.23	0.00	0.27	0.67	0.40
9	0.00	0.33	1.04	0.20	0.06	0.30	0.93	0.20
10	0.07	0.21	0.59	0.29	0.04	0.31	0.81	0.25
Average	0.029	0.33	0.90	0.23	0.032	0.22	0.65	0.23

**Table 3:** HTS room-temperature/550 °C high-temperature uniaxial tensile properties reference.

Room Temperature				550 °C High Temperature			
Rm	Rp0.2	A	Z	Rm	Rp0.2	A	Z
MPa	MPa	%	%	MPa	MPa	%	%
1094	962.5	16.25	61.5	751.3	653	22	73.81

**Table 4:** *Chinese Aviation Standards Manual* specifies 1Cr10 technical standards.

Room Temperature				550 °C High Temperature			
Rm	Rp0.2	A	Z	Rm	Rp0.2	A	Z
MPa	MPa	%	%	MPa	MPa	%	%
1000–1140	≥880	≥12	≥40	≥830	≥740	19	70

## Data Availability

The original contributions presented in this study are included in the article. Further inquiries can be directed to the corresponding author.
